# Cardiovascular Responses to Eccentric Cycling Based on Perceived Exertion Compared to Concentric Cycling, Effect of Pedaling Rate, and Sex

**DOI:** 10.3390/ijerph21010059

**Published:** 2023-12-31

**Authors:** Victorien Faivre-Rampant, Mark Rakobowchuk, Nicolas Tordi, Laurent Mourot

**Affiliations:** 1 MPFRPV, Exercise Performance Health Innovation (EPHI) Platform, Université de Franche-Comté, F-25000 Besançon, France; victorien14@live.fr; 2 UMRS 1075—Mobilités: Vieillissement, Pathologie, Santé, COMETE, University of Normandie, F-14000 Caen, France; 3 Department of Automatics, Biocybernetics, and Robotics, Jozef Stefan Institut, SI-1000 Ljubljana, Slovenia; 4 Jožef Stefan International Postgraduate School, Jamova cesta 39, 1000 Ljubljana, Slovenia; 5 Department of Biological Sciences, Faculty of Science, Thompson Rivers University, Kamloops, BC V2C 0C8, Canada; 6 PEPITE, Exercise Performance Health Innovation (EPHI) Platform, Université de Franche-Comté, F-25000 Besançon, France

**Keywords:** individualization, sex difference, cardiovascular responses, eccentric, cycling

## Abstract

Interest in eccentric exercises has increased over the last decades due to its efficiency in achieving moderate–high intensity muscular work with reduced metabolic demands. However, individualizing eccentric exercises in rehabilitation contexts remains challenging, as concentric exercises mainly rely on cardiovascular parameters. To overcome this, perceived exertion could serve as an individualization tool, but the knowledge about cardiovascular responses to eccentric cycling based on perceived exertion are still scarce. For this purpose, the cardiorespiratory parameters of 26 participants were assessed during two 5 min bouts of concentric cycling at 30 and 60 rpm and two bouts of eccentric cycling at 15 and 30 rpm matched for rating of perceived exertion. With this method, we hypothesized higher exercise efficiency during eccentric cycling for a same perceived exertion. The results revealed significantly elevated heart rate and cardiac index at higher pedalling rates during concentric (*p* < 0.001), but not during eccentric cycling (*p* ≈ 1). Exercise efficiency was higher during concentric cycling (64%), decreasing with pedalling rate, while eccentric cycling exhibited increased work rates (82%), and increased by over 100% with higher pedalling rate. Hence, eccentric cycling, with lower cardiorespiratory work for the same perceived exertion, facilitates higher work rates in deconditioned populations. However, further studies are needed for effective individualization.

## 1. Introduction

Eccentric contractions occur when an external load exceeds the force generated by the muscles, resulting in active muscle lengthening [1]. During eccentric contractions, muscles absorb mechanical work and produce significant amounts of force with a low metabolic cost [2]. Interest in eccentric-based exercise has increased over the last decade as this modality shows many benefits in patient care by easily enabling performance of moderate–high intensity exercise at a reduced metabolic demand, which is ideal amongst the elderly or patients with chronic disease [3,4,5,6]. In fact, eccentric (ECC) cycling using both moderate–high intensity and low–moderate intensity can increase muscle mass and function with minimal muscle damage if an adaptation phase is incorporated [7,8]. In previous studies that examined differences between ECC and Concentric (CON) modalities at the same absolute work rate, heart rate (HR) and cardiac output ($\dot{Q}$) are lower during ECC compared to CON [9,10,11,12,13]. Similarly, the energy requirements reflected by oxygen uptake ($\dot{V}$O_2_) are typically 4-fold lower than during CON cycling [4,14]. Conversely, with exercises performed at the same absolute $\dot{V}$O_2_, HR and work rate are higher during ECC cycling [9,15,16]. Beyond submaximal responses, maximal exercise capacities are also modified during ECC cycling with $\dot{V}$O_2_ max and HRmax being lower whereas maximum work rates are higher [17,18]. All these differences make prescribing exercise intensities to individuals difficult since these eccentric cycling responses are more complex than the well-characterized concentric exercise responses. Thus, simply prescribing intensity based on a percentage of HR, maximal work rate, or rating of perceived exertion (RPE) is not possible [9]. Ensuring an appropriately prescribed intensity of ECC exercise is important since inappropriate intensities may increase injury risk or nullify any benefits to patient care. Hence, the importance of evaluating responses to ECC exercise.

Unaccustomed or over-exertion during ECC exercises causes muscle damage and delayed onset muscle soreness (DOMS), which decrease range of motion, and muscle force, predisposing patients to a transient increased fall risk [19,20]. It also induces inflammation [21] and delayed onset vascular stiffness [22], which can be deleterious with patients in cardiovascular rehabilitation as it increase cardiovascular risk [23] and reduces baroreflex function [24,25,26,27]. In a rehabilitation context, such outcomes should be avoided, and hence an adapted ECC exercise prescription is mandatory.

To improve the prescription of ECC exercise some researchers focused on RPE as a potential convenient tool to guide intensity recommendations [28,29]. However, this strategy needs to be re-evaluated, especially since ECC exercises may induce a lower RPE at the same absolute work rate when directly compared to concentric exercise [12,30]. Moreover, pedalling rate modulates external work when performing CON exercise and increase or decrease total energy expenditure [31,32]. To complicate comparison further, RPE are also influenced by pedalling rate [33]. However, whether pedalling rate influences the RPE when people perform ECC cycling is currently unknown.

For constant work rate CON exercise bouts, $\dot{V}$O_2_ is significantly greater at 90 revolution per minute (rpm) compared to 30 and 60 rpm [34], in accordance with recent investigations demonstrating increased $\dot{V}$O_2_ at higher pedal frequencies during constant-load CON sessions [35]. But, during ECC contractions, the central nervous system employs a different neural strategy to control skeletal muscles that require more planning [11] and programming the movement with recruitment of larger area of the cortex [36]. Moreover, slow speed seems to be favourably tolerated with ECC exercises [28,37]. Also, because shortening velocity determines the extent of ECC cycling-induced muscle damage and soreness [38], a slow speed in the rehabilitation context may avoid such undesired effects. In this way, 30 and 15 rpm are commonly used in clinical settings [28,29]. Taken together, pedal frequency may have distinct impacts on the physiological response to CON and ECC which require further investigation [28].

Another important question is whether there are sex differences in the response to continuous ECC exercise. For decades, cardiovascular diseases were seen as a male’s disease but, the prevalence of cardiovascular diseases amongst females is not lower than males [39], and specific research on females has been scarce and more needed [40]. At rest, there are no differences in HR, but with males having higher stroke volumes than females, they also generally exhibit higher $\dot{Q}$ and mean arterial pressure (MAP) [41,42]. The cardiovascular regulation of males follows a sympathetic predominance compared to females that tend to have higher vagal activity [43,44]. During CON exercise, in spite of HR which increase similarly, cardiovascular responses differ between males and females, mainly because of female sex hormones [45,46] that induce more vasodilation that reduces relative peripheral resistances (PR), cardiac output, and MAP during dynamic CON exercise in females [47,48].

However, with the slight exception of RPE, these differences have not been fully considered in studies involving the ECC modality [49]. Consequently, the purpose of this study was to compare the acute cardiovascular and metabolic responses during CON and ECC cycling matched for RPE at two pedalling rates considered as slow and normal. We also explored potential difference between males and females and cardiovascular parameters.

During ECC, if the modality works the same way as CON, exercise efficiency was expected to be greater at the normal pedalling rate compared to the slow pedalling rate independent of the sex of the participants. Furthermore, because of lower average in vascular resistance in females, we expected a lower $\dot{Q}$ and PR amongst females during ECC compared to CON exercises at the same RPE and pedalling rate.

## 2. Materials and Methods

### 2.1. Study Population

Thirty participants were screened prior to testing and the exclusion criteria included: smoking, currently taking medication, and presence of apparent cardiovascular or metabolic disease. Participants were physical active (2 to 3 times per week of between 4 to 6 h). They had not participated in other activities involving ECC muscle actions over the past 6 months prior to the beginning of the study. Running was an acceptable routine physical activity for participants (i.e., not an exclusion criteria), except for when it involved prolonged downhill running. Written informed consent was obtained from each participant. In the end, 4 participants were excluded (3 due to exclusion criteria, 1 due to a dysfunction of our data acquisition system), and 26 healthy participants (13 females, 27.4 ± 8.3 years-old, BMI 22.9 ± 5.7 kg/m^2^, 61.1 ± 17.9 kg, height 1.63 ± 0.05 m; 13 males, 28.2 ± 8.9 years-old, BMI 23.5 ± 2.9 kg/m^2^, 74.8 ± 11.5 kg, height 1.78 ± 0.06 m) participated in this study. All procedures were performed between January and June 2016 in accordance with the ethical standards of the institutional research committee (Comité de Protection des Personnes Est I; number 2014-A00501-46) and complied with the Declaration of Helsinki [50].

### 2.2. Procedures

All sessions took place in the Exercise Performance Health Platform in Besançon after the validation of the study by Ethics Committee (University of Franche-Comté, France) in a temperature-, pressure-, and humidity-controlled room (20 °C, 765 mmHg, 50% of relative humidity). Participants visited the laboratory on two occasions: a baseline visit and one experimental session during which they completed different bouts of ECC or CON cycling.

Baseline visit: anthropometry and inclusion criteria.

Inclusion and exclusion criteria as well as anthropometric characteristics were determined by standard medical examination. Body weight was measured with a digital scale (resolution 0.1 kg; Seca 719, Hamburg, Germany) and barefoot standing height was assessed to the nearest 0.1 cm with a wall-mounted stadiometer (Seca 222, Hamburg, Germany).

### 2.3. Experimental Session

The experimental session was divided into two phases. The first phase comprised two 5 min bouts of CON cycling exercise performed at 30 (CONsl, for slow speed Concentric) and 60 rpm (CONnorm, for normal speed concentric) [51]. This first phase constituted a warm-up, which is why the order between the 2 CON bouts was not randomised. Equally, while the subjects were not familiar with perceived exertion and as the ECC intensity was based on the CON one, it also constituted a CON baseline for perceived exertion. The intensity was determined based upon the subjective perception of the participant: the power was adjusted to a level that the participant referred to as a light to moderate exercise RPE based on the CR-100 Borg scale [52]. The second phase comprised two 5 min bouts of ECC cycling exercise, and the instruction was to follow the same RPE as felt during CON; the two ECC bouts were performed in random order to avoid an order effect during the ECC phase. These bouts were performed at the same RPE (light to moderate on the CR-100 Borg scale), at a pedalling frequency of 15 rpm (ECCsl, for slow speed eccentric) and 30 rpm (ECCnorm, for normal speed eccentric) [24,37]. According to previous studies, 5 min bouts of exercises are sufficient to examine these responses [24,38] since a steady state is achieved within this timeframe. Data were measured during all 5 min bouts, but only the last minute of each bout was analysed since this was at steady state.

### 2.4. Ergometers

The ECC ergometer was a semi-recumbent prototype (developed by University Hospital Center of Dijon and TMS) driven by an asynchronous motor which enabled different pedalling frequencies [15,16,28]. To avoid hemodynamic differences due to position during CON cycling, we also used a semi-recumbent ergometer (Excite+—recline, Technogym SpA, Cesean, Italy). The CON cycling consists of a classic pedalling activity on a semi-recumbent ergometer. The ECC cycling, in contrast, is less familiar. During this modality, the ergometer induces a backward pedalling movement at which the subject needs to resist. In both cases, the exercise was a continuous alternation in quadriceps contractions during half of a rotation. Only the rhythm, the contraction modality, and the neuromuscular pattern change between the conditions.

### 2.5. Cardiovascular and Respiratory Evaluations

Exercise responses were recorded continuously at rest (baseline) and throughout exercise. Breath-by-breath pulmonary gas exchange and ventilation were continuously measured using metabolic cart (Metalyzer 3B-R3 system; Cortex Biophysics, Leipzig, Germany). Calibration was performed before each test according to manufacturer instructions using precision gases (CO_2_ = 5% and O_2_ = 15%) and a 3 L volume syringe. HR was measured using a 3-lead electrocardiograph. Signals were digitized and stored using a data acquisition system (PL3008 PowerLab 8/30, ADInstruments, Colorado Springs) and software (LabChart 7, ADInstruments). $\dot{Q}$ and MAP were estimated non-invasively using the Model Flow method (Finometer Pro; Finapres Medical Systems, Arnheim, The Netherlands). This device uses a photoplethysmographic sensor and cuff placed on the third finger of the right hand. It was validated and used previously at low exercise intensities similar to those used in the present study [15,53]. Additionally, arterial pressure was measured in the right arm by an electro-sphygmomanometer (Omron Healthcare, Kyoto, Japan) to calibrate the continuous arterial blood pressure measurements. Based on the relationship between variables, PR was calculated from the ratio of MAP and $\dot{Q}$ values according to the Ohm’s law [54]. Meanwhile, exercise efficiency (kcal/W) was calculated from $\dot{V}$O_2_, $\dot{V}$CO_2_, and work rate during the steady state using the Jeukendrup’s equation for energy expenditure, which is supposed to be the more accurate according to Kipp et al. in 2018, and this equation assumes negligible contribution of protein oxidation [55,56]:

(1) $$Energy\;expenditure\;(kcal/min)=(0.575\times \dot{V}{\mathrm{CO}}_{2}+4.435\times \dot{V}O_{2})$$

Exercise efficiency (kcal/W) = energy expenditure/work rate
(2)

### 2.6. Data and Statistical Analyses

All data are presented as means ± standard deviation (SD), the level of significance was set at *p* < 0.05 for all statistical tests, and the effect size was calculated using Cohen’s D. The data were evaluated for normality of distribution using the Shapiro–Wilk test, and when violated, data were log transformed. A 3-way repeated measures ANOVA was performed to identify significant differences and interactions between factors (modality, pedalling rate, sex), which were further examined using Bonferroni-corrected post hoc tests. The minimum required sample size for investigating “repeated measures, within factors” was calculated using the results of two previous studies [10,15]. Using these data, an effect size (f) between 0.292 and 1.305 (equating to an effect size (d) between 0.583 to 2.610) for the comparison between modalities and cardiac output was computed. Assuming an α of 0.05 and β of 0.95, 27 participants would provide sufficient power to detect a statistical difference of a similar magnitude (G*Power Version 3.1.9.2). To account for drop-out, 30 participants were recruited, and at the end only 26 subjects lasted for the data analysis but the statistical power was verified a posteriori using G*Power. For all significant differences the power was higher than 0.8 (G*power 3.1.9.6, Kiel University, Germany).

## 3. Results

### 3.1. Rating of Perceived Exertion

Overall, RPE was not significantly different between CON and ECC independent of the pedalling rate (CON = 15.37 ± 7.12 ECC = 15.42 ± 6.97, *p* = 0.965), but it was lower during exercises with a low pedalling rate compared to a higher pedalling rate independent of the modality (main effect for pedalling rate: *p* < 0.001, d = 0.657).

Concerning sex differences, RPE was not different between males and females during exercise bouts (*p* = 0.467). There were no interactions between parameters except a sex × modality × pedalling rate (*p* = 0.014). Upon post hoc comparison, the only RPE difference that exist was amongst females when they performed CONsl versus CONnorm cycling (*p* < 0.001, d = 1.218).

### 3.2. Anthropometrics

With males being significantly bigger and taller than women (1.78 ± 0.06 vs. 1.63 ± 0.05; *p* < 0.001), cardiac output is corrected with body size to have the cardiac index.

### 3.3. Cardiovascular Responses

**Figure 1:**
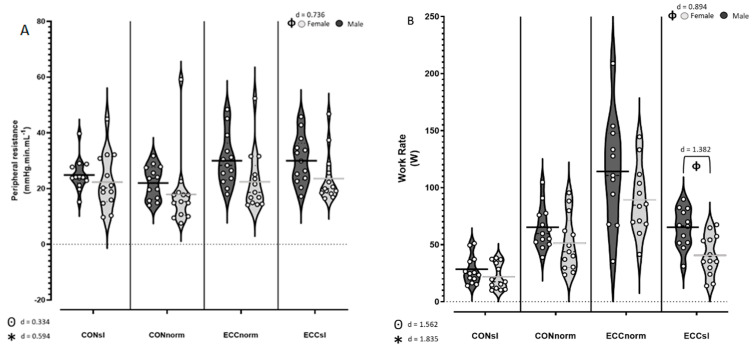
(**A**) Violin plot of peripheral resistances for males (black) and females (grey) during concentric (CON) and eccentric (ECC) cycling at slow (sl) and normal (norm) pedalling rate. (**B**) Work rate for males and females during CON and ECC cycling at slow and normal pedalling rate. * indicates a modality effect, ⊙ indicates a pedalling rate effect, and ϕ indicates a sex effect. Cohen’s D was used to calculate effect sizes and they are interpreted as: 0 < d < 0.2 Trivial difference, 0.2 < d < 0.4 Very-low difference, 0.4 < d < 0.6 Low difference, 0.6 < d < 0.8 Moderate difference, 0.8 < d < 1.2 High difference, 1.2 < d < 2 Very-high difference, 2 < d < 4 Enormous difference.

Table 1 shows the cardiovascular responses to cycling during the different modalities and cadences. HR was not significantly different between males and females, while cardiac index was higher in females and MAP higher in males independent of the pedalling rate. However, it seems that increasing pedalling rate also increases the cardiac index in women but not in men, independent of the cycling modality. Furthermore, PR was higher in males compared to females (*p* = 0.032, d = 0.736) (Figure 1A). At slow pedalling rates, HR was not significantly different during CON and ECC (*p* ≈ 1) as well as $\dot{Q}$ (*p* ≈ 1). Furthermore, HR and cardiac index were significantly elevated at higher pedalling rates during CON (Cardiac index; CONno > CONsl, *p* < 0.001, d = 1.122) but not during ECC cycling (Cardiac index; ECCno = ECCsl, *p* ≈ 1). MAP was not significantly affected by modality nor by pedalling rate. PR was significantly higher during ECC than CON (*p* < 0.001, d = 0.594) (Figure 1A). All other statistical analyses showed no differences or interactions amongst cardiovascular parameters (Table 1).

### 3.4. Metabolic Responses

**Figure 2:**
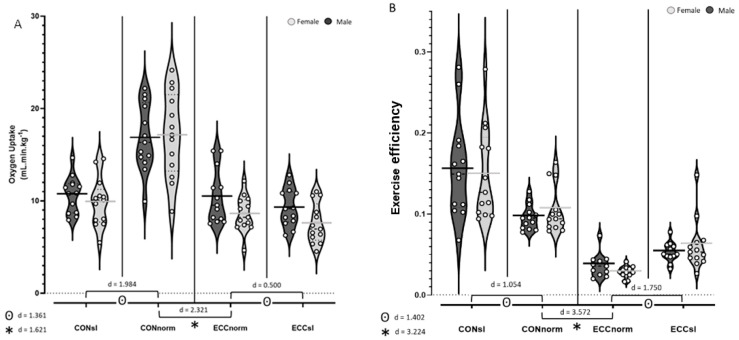
(**A**)Violin plot analysis of $\dot{V}$O_2_ for males and females during CON and ECC cycling at slow and normal pedalling rate. (**B**) Exercise efficiency for males and females during CON and ECC cycling at slow and normal pedalling rate. * indicates a modality effect, ⊙ indicates a pedalling rate effect. Cohen’s D was used to calculate effect sizes and they are interpreted as: 0 < d < 0.2 Trivial difference, 0.2 < d < 0.4 Very-low difference, 0.4 < d < 0.6 Low difference, 0.6 < d < 0.8 Moderate difference, 0.8 < d < 1.2 High difference, 1.2 < d < 2 Very-high difference, 2 < d < 4 Enormous difference.

As expected, post hoc analyses showed that $\dot{V}$O_2_ (Figure 2A) was higher during CON than ECC (*p* < 0.001, d = 1.579). Also, an interaction in modality × pedalling rate was apparent (*p* < 0.001) suggesting that $\dot{V}$O_2_ is substantially increased with pedalling rate when performing CON (CONno > CONsl, *p* < 0.001, d = 1.984), but only modestly increased by pedalling rate with ECC (ECCno > ECCsl, *p* = 0.002, d = 0.500, Figure 2A). Moreover, while exercise efficiency (Figure 2B) was higher during CON compared to ECC (*p* < 0.001, d = 3.224) and decreased with pedalling rate (*p* < 0.001, d = 1.402, Figure 2B), work rate was higher during ECC (*p* < 0.001, d = 1.562) and increased with pedalling rate (*p* < 0.001, d = 1.835, Figure 1B).

$\dot{V}$O_2_ and exercise efficiency were not significantly different between females and males, whereas work rate was significantly higher in males compared to females, especially during ECCsl (sex × modality × pedalling rate interaction; *p* = 0.048, Figure 1B).

## 4. Discussion

In this study, we explored cardiometabolic differences between CON and ECC cycling at different pedalling rates matched for the same RPE and a potential sex effect. Overall, there was no interaction between sex and modality, which suggests that differences between males and females during the ECC modality are similar to those we know exist during CON.

Moreover, and as expected, there was no difference in RPE between modalities nor between sexes, but a low pedalling rate impacted the metabolic rate and cycling efficiency, which suggests that with similar instructions to maintain the same RPE, people tend to expend less energy and are more efficient with a slow pedalling frequency.

During ECCsl, the work rate is equivalent to CONnorm suggesting that when participants self-selected exercise intensities based on RPE, ECCsl may be equivalent to CONnorm and may produce a similar absolute work rate. Interestingly, exercise efficiency is lower with ECC at the same RPE, and this effect is not sex dependent. Also, exercise efficiency decreases at faster pedalling rates, which has previously been shown up until participants reach an optimal pedalling rate during CON cycling [57]. During ECC, this optimal pedalling rate is still under investigation and depends on the considered parameter. Further studies are needed to determine the metabolically optimal pedalling rate during ECC [58].

Concerning sex differences, at a similar pedalling rate and RPE, work rate was higher during ECC than CON independent of sex, while males’ work rate was slightly higher than females during both CON and ECC cycling.

As expected, $\dot{V}$O_2_ was lower during ECC than CON [9] and was increased with pedalling rate. Interestingly, pedalling rate impacted $\dot{V}$O_2_ more during CON (d = 1.984) than ECC (d = 0.500) cycling. As previously demonstrated by Peñailillo et al. (2017) [59], this may be explained by a lower oxygen requirement due to a lower muscle activity during ECC cycling at the same work rates [59]. In this way, increasing the pedalling rate and so with it the work rate will increase the oxygen requirement relatively more during CON compared to ECC. Similar to work rates, $\dot{V}$O_2_ did not depend on sex.

Finally, the responses in $\dot{V}$O_2_, work rate, and cycling efficiency at a same RPE suggest that ECC modality may be more manageable for extremely deconditioned populations and that slow pedalling rates may be even more effective and well tolerated.

Concerning cardiovascular responses, MAP responses were not dependent on modality or pedalling rate, while cardiac index increased with pedalling rate but only during CON and not ECC. Equally, if the modality is not taken into account, it seems that an increase in pedalling rate induce a significant increase in cardiac index for females but not for males. Moreover, independent of the modality or pedalling rate, MAP was higher in males resulted in higher PR amongst males.

Those two observations were reflected in a higher PR during ECC. We also noted that PR decreased with increasing pedalling rate under CON cycling condition but not during ECC cycling. Similarly, the almost twice higher work rate during ECC could explain the higher PR with this modality. Additionally, an interaction between modality and pedalling rate may exist (*p* = 0.057), but more studies are needed to confirm this. Interestingly, work rate was higher in males, at a similar RPE, which likely accounts for their higher PR.

The increase in HR following the increase in pedalling rate was present only during CON which agrees with previous studies [9].

Other work has suggested there are no sex differences in HR during peak exercise with both CON and ECC modalities [42,47]. As such, our findings followed this same HR response, which was similar amongst males and females during light–moderate intensity cycling exercises. This response in HR reflects a lower cardiac work during ECC than CON when exercising at the same RPE, but ECC exercises seems to increase PR especially amongst males which should be considered in the context of cardiac rehabilitation. Further research within the cardiac rehabilitation population would be worthwhile.

### Strenghts and Limitations

The main strength of this study is the investigation of both pedalling rates and sex effect for a same perceived exertion on cardiorespiratory responses during ECC cycling compared to CON, as well as the use of different pedalling rate between modalities to take into account the neuromuscular differences. This is also the main limitation of this study, and considering a larger range of pedalling rates with also equal pedalling rates could support our previous argument and give further information about the optimal pedalling rate during ECC, with the aim of improving its individualization based on perceived exertion in a rehabilitation context.

## 5. Conclusions

The main goal of this study was not to focus on sexual differentiation, and in this way, menstrual phase and hormonal contraceptive methods amongst female participants were not controlled. However, since both conditions (ECC and CON) and pedalling rates were tested within a short period of time on the same day, the impact on the cardiovascular responses of these factors would be minimal. However, we cannot discount the possibility that hormonal influences may exist and would be worth examining in subsequent studies [40].

In relation to the application of ECC modalities in the cardiac rehabilitation context, the focus of these programs is often on improving cardiorespiratory function, and although ECC is an effective method to improve muscular strength and endurance, this modality may not sufficiently stimulate the cardiorespiratory systems. In fact, this type of training may induce increased PR, especially for males, and this may not be ideal. Hence, further studies within older adults attending rehabilitation centres seem to be needed.

In the same way, the impact of pedalling rate on physiological parameters during ECC cycling continues to be insufficiently understood, and the optimal pedalling rate has not been determined.

Currently, studies exploring pedalling rate during ECC cycling have only examined muscular parameters [34,60].

Finally, the ECC modality does not induce specific sex differences on cardiorespiratory parameters, and so, knowledge of sex differences in CON cycling could be used during ECC cycling in rehabilitation and for future studies.

## Figures and Tables

**Table 1:** Comparison of cardiovascular response of men and women between concentric and eccentric cycling at slow and normal speed.

Parameters		Data				Simple Effect			Interactions			
		CONsl	CONno	ECCsl	ECCno	Modality *	Speed ⊙	Sex ϕ	Modality × Speed	Modality × Sex	Speed × Sex	Modality × Speed × Sex
Heart Rate	♀	92.56 ± 9.48	113.74 ± 19.22	93.67 ± 9.92	96.72 ± 8.78	***p* < 0.001** **d = 0.590**	***p* < 0.001** **d = 0.790**	*p* = 0.096 d = 0.590	***p* < 0.001** **Increase only on CON while increasing speed**	*p* = 0.955	*p* = 0.299	*p* = 0.237
	♂	88.32 ± 10.93	102.85 ± 13.72	86.10 ± 14.03	89.65 ± 16.51							
Cardiac Index L/min/m^2^	♀	2.729 ± 0.640	3.945 ± 1.348	2.826 ± 0.737	3.062 ± 0.950	***p* = 0.004** **d = 0.553**	***p* < 0.001** **d = 0.662**	***p* = 0.002** **d = 1.081**	***p* < 0.001** **Increase only on CON while increasing speed**	*p* = 0.811	***p* = 0.035 Increase only on CON while increasing speed**	*p* = 0.108
	♂	2.285 ± 0.395	2.2792 ± 0.644	2.045 ± 0.497	2.118 ± 0.478							
Mean Arterial Pressure mmHg	♀	93.52 ± 25.32	99.63 ± 32.66	101.57 ± 16.19	100.91 ± 18.04	*p* = 0.127 d = 0.291	*p* = 0.075 d = 0.184	***p* = 0.036** **d = 0.696**	*p* = 0.285	*p* = 0.547	*p* = 0.495	*p* = 0.729
	♂	105.87 ± 14.07	113.26 ± 14.86	111.66 ± 15.07	115.73 ± 16.66							
Peripheral Resistances	♀	22.62 ± 10.06	18.17 ± 13.04	23.82 ± 9.00	22.72 ± 10.81	***p* < 0.001** **d = 0.594**	***p* = 0.009** **d = 0.334**	***p* = 0.032** **d = 0.736**	*p* = 0.057 Decrease only on CON while increasing speed	*p* = 0.678	*p* = 0.202	*p* = 0.621
	♂	24.94 ± 5.79	22.28 ± 5.92	30.23 ± 8.69	30.14 ± 9.30							

* Data are mean ± standard deviation; modality show significant difference with * if *p* < 0.05; pedalling rate show significant difference with ⊙ if *p* < 0.05; sex show significant difference with ϕ if *p* < 0.05; Cohen’s D expresses effect size and is interpreted as: 0 < d < 0.2 Trivial difference, 0.2 < d < 0.4 Very-low difference, 0.4 < d < 0.6 Low difference, 0.6 < d < 0.8 Moderate difference, 0.8 < d < 1.2 High difference, 1.2 < d < 2 Very-high difference, 2 < d < 4 Enormous difference.

## Data Availability

Original data will be made available to any qualified researcher upon request.
